# Halophyte-Specific Rhizosphere Effects Drive the Differentiation of Microbial Community Assembly in a Desert-Grassland Salt Marsh

**DOI:** 10.3390/microorganisms14030635

**Published:** 2026-03-11

**Authors:** Rong Wang, Jinpeng Hu, Jialu Li, Zixuan Chen, Bahetijiang Ayala, Xigang Liu, Peng Kang, Yaqing Pan

**Affiliations:** 1 Xinjiang Laboratory of Lake Environment and Resources in Arid Zone, College of Geographic Science and Tourism, Xinjiang Normal University, Urumqi 830054, China; wangrong081624@163.com (R.W.); lijialu202306@126.com (J.L.); chenzixuan0710@163.com (Z.C.); meiheban6823@163.com (B.A.); liuxigang20@mails.ucas.ac.cn (X.L.); kangpeng@xjnu.edu.cn (P.K.); 2 College of Pastoral Agriculture Science and Technology, Lanzhou University, Lanzhou 730000, China; 17693044924@163.com

**Keywords:** desert-grassland salt marsh, halophytes, rhizosphere microbiome, microbial co-occurrence network, network complexity

## Abstract

Arid salt marsh ecosystems endure chronic water scarcity and high salinity stress, with the stability of their functions inextricably linked to the pivotal role of the rhizosphere microenvironment of halophytes. This study focused on three typical halophytes (*Kalidium cuspidatum, Nitraria tangutorum, Reaumuria soongarica*) in the Jiantan wetland, and deeply explore how these halophytes differently regulate the soil microenvironment through the rhizosphere effect. The results showed that the rhizosphere soil of *Kalidium cuspidatum* had higher pH, Na^+^, and K^+^ contents, while the rhizosphere soil of *R. soongarica* had higher total carbon, soil organic carbon, alkali-hydrolyzable nitrogen, and microbial biomass. Microbial community analysis revealed that rhizosphere soil of fungal diversity was significantly higher in *K. cuspidatum* than in *R. soongarica*, with distinct differences in bacterial and fungal community structures. These differences were closely associated with factors such as Na^+^, Olsen phosphorus, microbial biomass carbon and alkali-hydrolyzable nitrogen. Among the dominant phyla, Proteobacteria and Ascomycota predominate, with *Desulfobacterota* and *Mortierellomycota* exhibiting the highest explanatory power (>48%) for physicochemical property variations. The microbial network of rhizosphere soil of *R. soongarica* has the highest complexity (with 633 nodes and 3300 edges), but the proportion of positive correlation edges was the lowest (21.58%). Structural equation modeling indicates that soil physical properties indirectly influence network complexity by negatively regulating chemical properties and microbial biomass, while microbial diversity had a direct positive effect on dominant phylum composition and network complexity. This study elucidated the differentiated adaptive strategies of rhizosphere microenvironment-microbe interactions in halophytes, providing a theoretical basis for wetland ecological restoration.

## 1. Introduction

In the salt marsh ecosystems of arid regions, halophytes are not evenly distributed but follow strict ecological gradients, presenting distinct band-like or patchy patterns [1]. From the core area of the salt marsh to the ecotone between water and land, with changes in soil salinity, moisture conditions, and groundwater level, there are plant species with different salt-tolerant levels distributed successively, such as *Phragmites australis*, *Kalidium cuspidatum, Nitraria tangutorum, Reaumuria soongarica,* and *Tamarix chinensis.* Together, they form a rare green living area in an arid region [2,3]. This distribution pattern is not only the result of plant adaptation and selection to environmental stress but also the core that maintains the stability of this ecosystem [4]. These halophytes fix quicksand and saline soil through their dense root systems, effectively mitigating soil wind and water erosion, and serve as a frontline barrier against desertification [5]. Its vast life forms can also fix carbon in the atmosphere, becoming an important carbon sink[4,5,6]. Therefore, the survival and distribution of halophytes in salt marshes in arid regions directly determine the structure, function, and stability of this ecosystem. In-depth research on these species is the scientific basis for ecological restoration and biodiversity conservation.

The rhizosphere microorganisms of plants serve as a link between plants and soil, and together they form a complex and dynamically balanced rhizosphere micro-ecosystem [7]. Compared with common plants, the rhizosphere of halophytes forms a highly specialized “stress screening environment”, thereby enriching a group of microbial groups with excellent salt tolerance and growth-promoting functions[8]. Bacterial communities, such as *Bacillus* and *Pseudomonas*, directly assist plants in resisting salt stress and improving their nutritional status through nitrogen fixation, phosphorus dissolution, secretion of osmotic regulatory substances, and ACC deaminase [9,10]. Fungal communities (especially arbuscular mycorrhizal fungi) play the role of “underground network engineers”, and their vast mycelial networks greatly expand the absorption range of the root system, helping plants obtain water and weakly diffused nutrients such as phosphorus more efficiently in the vast saline soil [11,12]. This rhizosphere micro-ecosystem, dominated by halophytes and featuring the precise collaboration of bacteria and fungi, is the core for its successful survival and ecological restoration functions in harsh saline environments and provides a crucial biological basis for the development of saline-alkali land management strategies based on plant-microbial consortia [13,14].

Bacteria and fungi in the plant rhizosphere do not exist in isolation. Through complex interactions, they form a sophisticated “bacterial–fungal co-occurrence network” [15]. This network was constructed based on high-throughput sequencing and bioinformatics analyses. By statistically analyzing the co-occurrence and mutual exclusion relationships among species, the internal organizational structure and ecological connections of microbial communities are visually revealed [16]. Microbial network complexity is directly related to the functional stability and environmental adaptability of the community [17]. In adverse environments, such as those inhabited by halophytes, a more connected and stable co-occurrence network usually indicates that the microbial community can collaborate more efficiently to help the host plant resist salt stress and obtain nutrients, thereby demonstrating stronger ecological resilience [18,19]. Therefore, analyzing this co-occurrence network is a core link in understanding the assembly rules of the rhizosphere microbial community, its functional synergy, and the mechanism of its impact on plant health.

This study selected three representative species, such as *K. cuspidatum, N. tangutorum* and *R. soongarica.* The aim of this study was to systematically reveal the composition, diversity, and structural characteristics of bacterial and fungal communities in the rhizosphere soil. This study will utilize high-throughput sequencing technology and combine the physicochemical parameters of the rhizosphere to explore the differences in the microbial community structure in the rhizosphere of different halophytes, with the aim of characterizing the associations between halophytes and rhizosphere microorganisms in salinized habitats by analyzing bacterial–fungal co-occurrence networks.

## 2. Materials and Methods

### 2.1. Overview of the Study Area and Collection of Rhizosphere Soil

**Figure 1:**
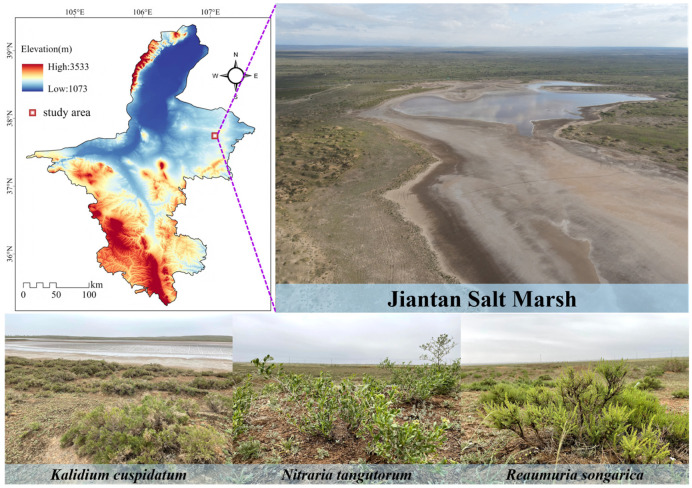
Sampling sites in the Jiantan wetland in Yanchi County, Ningxia.

The study site was located in the Jiantan wetland in Yanchi County, central Ningxia. It belongs to a typical desert steppe salt marsh wetland ecosystem, with an altitude of 1398 m, an average annual temperature of 7.8 °C, an annual rainfall of approximately 280 mm, and an evaporation of over 2000 mm [20]. The wetland extends from the water-land ecotone to the desert steppe, mainly distributing plants such as *Kalidium cuspidatum*, *Nitraria tangutorum*, *Reaumuria soongarica*, *Sophora alopecuroides*, and *Stipa caucasica.* We investigated three halophytes, *K. cuspidatum*, *N. tangutorum* and *R. soongarica* (Figure 1). The soil attached to the roots of the plants was shaken and collected and defined as rhizosphere soil [21]. A total of 18 samples were collected from the rhizosphere soil of each halophytic plant, with three samples serving as one sample, totaling six samples. Soil samples were transported to the laboratory using a mobile refrigerator. After screening in the laboratory, the physical and chemical parameters and microbial biomass of the soil samples were determined, and soil DNA was extracted.

### 2.2. Determination of Physical and Chemical Properties of Soil

The soil water content (SWC) was determined using the weighing method. After the samples were air-dried, deionized water (soil/water = 1/2.5) was added and stirred evenly, and the pH value and electrical conductivity (EC) of the soil were measured using a pH meter and a conductivity meter [22]. The soil Na^+^ and K^+^ contents were determined using a flame photometer (FP6410, Shanghai INESA Analytical Instrument Co., Ltd., Shanghai, China) [23]. After the air-dried soil sample was sieved (0.15 mm), the total soil carbon (TC) and inorganic carbon (TIC) contents were determined using the TOC-VSeries SSM-5000A determination component [24]. The value of TC minus TIC is soil total organic carbon (TOC). Similarly, the total nitrogen content (TN) in the soil was determined using an elemental analyzer, and the total phosphorus (TP) content in the soil was determined using the sulfuric acid-molybdenum-dysprosium anticolorimetric method [25]. Soil alkali-hydrolyzable nitrogen (AN) was determined using the alkali-hydrolyzed diffusion method, and available phosphorus was extracted and determined using the sodium bicarbonate method [22]. After the soil samples were extracted with NaHCO_3_, the Olsen phosphorus content (OP) was determined using molybdenum blue colorimetry [26].

### 2.3. Determination of Soil Microbial Biomass

Fresh soil samples were fumigated with chloroform (CHCl_3_), and the fumigated and unfumigated soils were extracted with K_2_SO_4_ to determine the dissolved organic carbon (DOC) and total nitrogen in the soil, respectively [27,28]. The phosphorus content of soil microorganisms was determined using the molybdenum blue colorimetric method [29].

(1) $$Microbial\;biomass\;carbon\left(MBC\right)=\frac{{DOC}_{fumigation}-{DOC}_{non-fumigation}}{0.45}\;$$

(2) $$Microbial\;biomass\;nitrogen\left(MBN\right)=\frac{{TN}_{fumigation}-{TN}_{non-fumigation}}{0.54}\;$$

(3) $$Microbial\;biomass\;phosphorus\left(MBP\right)=\frac{{TP}_{fumigation}-{TP}_{non-fumigation}}{0.40}\;$$

Among them, 0.45, 0.54, and 0.40 are the efficiency coefficients of fumigation for extracting carbon, nitrogen, and phosphorus from microbial biomass, respectively.

### 2.4. Soil DNA Extraction and High-Throughput Sequencing

Weigh fresh soil samples (1.0 g), add the extract (100 mmol·L^−1^ Tris-HCl, 100 mmol·L^−1^ EDTA, 200 mmol·L^−1^ NaCl, 1.0% PVP, 2.0% CTAB, pH 8.0), boil, cool and then centrifuge. The supernatant was extracted and added to (100 mmol·L^−1^ Tris-HCl, 200 mmol·L^−1^ NaCl, 2.0% SDS, pH8.0). After centrifugation, the supernatant was used for gel purification. Subsequently, the V5–V7 region of the bacterial 16S rRNA gene was amplified using primers 799F (5′-AACM GGAT TAGA TACC CKG-3′) and 1193R (5′-ACGT CATC CCCA CCTT CC-3′) [30]. PCR amplification of fungal ITS was performed using the primers ITS1F (5 ‘-CTTG GTCA TTTA GAGG AAGT AA-3′) and ITS2 (5 ‘-GCTG CGTT CTTC ATCG ATGC-3′) [31]. After PCR amplification, the samples were sequenced using the Illumina MiSeq PE300 platform. The original data were spliced using FLASH (V1.2.11) software [32]. Cluster analysis of operational taxonomic units (OTUs) was conducted using Usearch software (similarity = 97%) [33]. Subsequently, the α-diversity indices (Shannon and ACE) of bacterial and fungal communities were obtained using QIIME (Version 1.9) software [34]. The original data of bacteria and fungi in the soil samples of this study were submitted to the NCBI database (PRJNA1199940 and PRJNA1200139).

### 2.5. Data Analysis

All data were statistically analyzed using analysis of variance (ANOVA). Mantel tests for microbial diversity and physicochemical parameters were conducted using the “linkET” package in R software (V4.1) [35], and the data were visualized through the “ggplot2” package [36]. To further reveal the differences in rhizosphere microbial communities among different halophytes, non-metric multidimensional scaling analysis (NMDS) was used for visual display [37]. The “pheatmap” package was used to conduct Spearman analysis on the correlations between the data. The relative abundance (>1%) of the dominant phylum in the rhizosphere of halophytes was visualized using the “ggalluvial” package. Random forest regression analysis was conducted using the “randomForest” package to evaluate the significance of the dominant phyla in each microbial community to physicochemical properties and microbial biomass [38]. This study further identified the dominant bacterial genera in the rhizosphere of halophytes (top 10 in relative abundance) and conducted a variation partitioning analysis of the effects of soil physical behavior, chemical properties, and microbial biomass on the dominant bacterial genera using the “vegan” package [39].

The bacterial–fungal co-occurrence network in the rhizosphere provides direct evidence of the strength of the relationships between different taxa. In this study, Spearman analysis was conducted on the correlation of bacterial–fungal OTUs in the rhizosphere soil of three halophytes (|r| > 0.9, *p* < 0.01), and a matrix was constructed. The data was visualized using Cytoscape software (3.7.1). The “igraph” package was also used to extract relevant data from the subnetwork [40]. PCA was conducted on nodes, edges, average density, Transitivity, Diameter, and Average path length, and PCA1 was listed as the network complexity index (NCI) [41,42]. The linear regression relationship between dominant phyla and network complexity was demonstrated using the “ggplot2” package, and using “plspm” package to construct the partial least squares path model (PLS-PM) [43].

## 3. Results

### 3.1. Differences in Physical and Chemical Properties of Rhizosphere Soil of Halophytes

In the Jiantan wetland, the pH, SWC, Na^+^, and K^+^ contents in the *K. cuspidatum* rhizosphere soil were all higher than those in the *N. tangutorum* and *R. soongarica* rhizosphere soils. The Na^+^ content was 3.14 times and 68.65% higher than that of the *N. tangutorum* and *R. soongarica* rhizosphere soils, and the K^+^ content was 8.87 times and 1.06 times higher (*p* < 0.05). *R. soongarica* rhizosphere soil has a relatively high content of TC, TOC, and AN, which are 93.11%, 1.17 times, and 88.89% higher than those of *K. cuspidatum*, respectively. In addition, the MBC, MBN, and MBP content were higher in the *R. soongarica* rhizosphere soil, which were 31.45%, 25.66%, and 25.46% higher than those of *K. cuspidatum*, respectively (*p* < 0.05) (Table 1).

### 3.2. The Diversity of Rhizosphere Microorganisms of Halophytes

**Figure 2:**
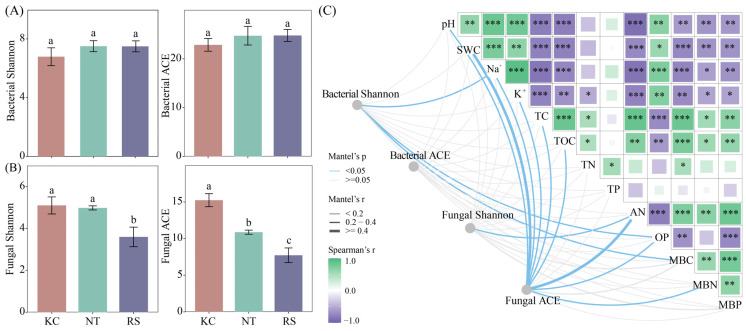
The alpha diversity (**A**,**B**) of bacteria and fungi in the rhizosphere soil of halophytes and its correlation with physical and chemical properties (**C**). Different lowercase letters indicate significant differences. (*** *p* < 0.001, ** *p* < 0.01, * *p* < 0.05).

In the Jiantan wetland, there was no significant difference in the Shannon and ACE of the rhizosphere bacterial communities of *K. cuspidatum*, *N. tangutorum*, and *R. soongarica***.** The Shannon and ACE fungal communities in the rhizosphere of *K. cuspidatum* were significantly higher than those in the rhizosphere of *R. soongarica* (*p* < 0.05) (Figure 2A,B). Mantel’s test showed that Shannon’s bacterial diversity was significantly correlated with Na^+^, OP, and MBC. Fungal Shannon and AN had a significant correlation. Fungal ACE was strongly correlated with pH, SWC, TOC, AN, OP and MBN (*p* < 0.05) (Figure 2C).

**Figure 3:**
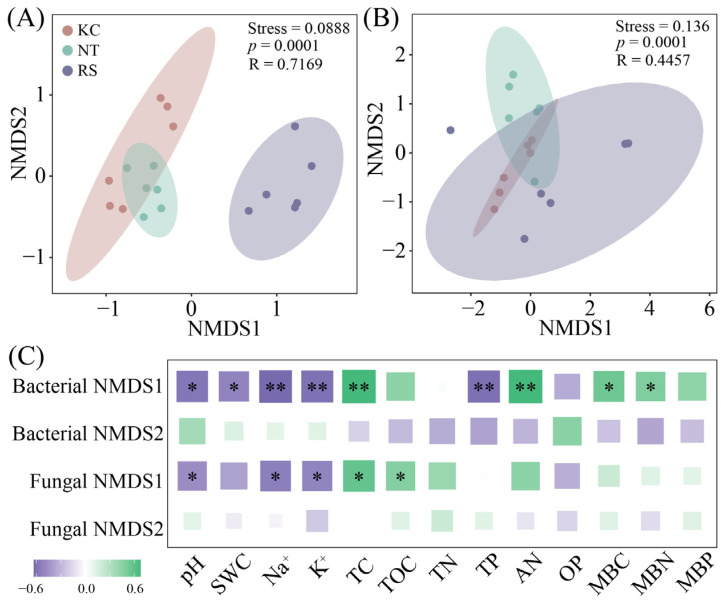
The beta diversity (**A**,**B**) of bacteria and fungi in the rhizosphere soil of halophytes and its correlation with physical and chemical properties (**C**). (** *p* < 0.01, * *p* < 0.05).

Further analysis revealed that both the rhizosphere bacterial (Stress = 0.0888, *p* = 0.001) and fungal (Stress = 0.136, *p* = 0.001) communities of halophytes showed certain differences (Figure 3A,B). Based on the analysis of physicochemical properties, bacterial NMDS1 showed a significant positive correlation with pH, SWC, Na^+^, K^+^, and TP, and a significant negative correlation with TC, AN, MBC, and MBN. Fungal NMDS1 was positively correlated with pH, Na^+^, and K^+^, and negatively correlated with TC and TOC (*p* < 0.05) (Figure 3C).

### 3.3. The Community Structure of Rhizosphere Microorganisms of Halophytes

**Figure 4:**
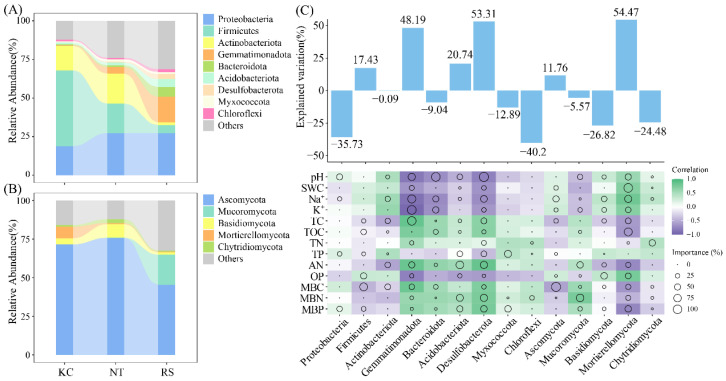
Relative abundance of dominant bacterial and fungal phyla (**A**,**B**) in the rhizosphere soil of halophytes and the random forest analysis with physicochemical properties (**C**).

Among the rhizosphere bacterial communities of the three halophytes, *Proteobacteria*, *Firmicutes*, *Actinobacteriota*, and *Gemmatimonadota* were the dominant phyla (Figure 4A), whereas *Ascomycota*, *Mucoromycota*, and *Basidiomycota* were the dominant phyla of the rhizosphere fungal community (Figure 4B). Further random forest analysis revealed that among the rhizosphere bacteria, *Desulfobacterota* (53.31%) and *Gemmatimonadota* (48.19%), And *Mortierellomycota* (54.47%) among rhizosphere fungi had a higher explanatory rate for the changes in rhizosphere physicochemical properties (*p* < 0.05) (Figure 4C).

**Figure 5:**
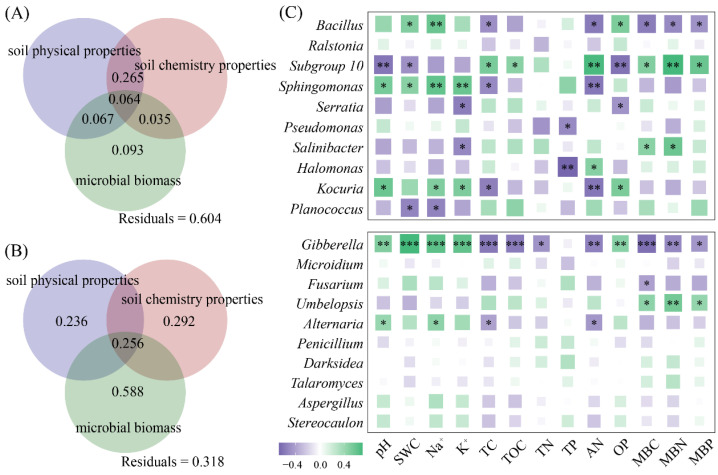
Variations in physical properties, chemical properties, and microbial biomass in the rhizosphere soil of halophytes (**A**,**B**), as well as the correlation between the top 10 genera and the physicochemical properties (**C**). (*** *p* < 0.001, ** *p* < 0.01, * *p* < 0.05).

We further selected the top 10 dominant bacterial and fungal genera based on their relative abundance (Figures S1 and S2). Variation decomposition revealed that the physical properties, chemical characteristics, and microbial biomass of the halophyte rhizosphere soil jointly explained 39.6% of the changes in the dominant genera of rhizosphere bacteria (Figure 5A) and had an explanation rate of 68.2% for the changes in the dominant genera of rhizosphere fungi (Figure 5B). The genera and physicochemical properties of rhizosphere bacteria, such as *Bacillus*, *Subgroup 10*, *Sphingomonas*, and *Kocuria*, were strongly correlated, and the rhizosphere fungi *Gibberella* and *Alternaria* were strongly correlated with changes in pH, Na^+^, TC, and AN (Figure 5C).

### 3.4. The Bacterial–Fungal Co-Occurrence Network in the Rhizosphere of Halophytes

**Figure 6:**
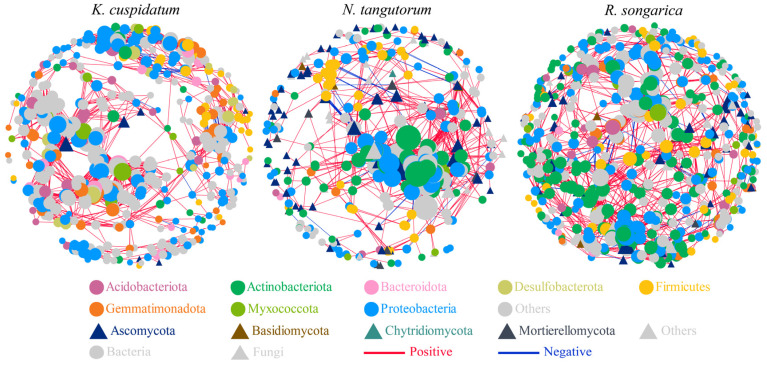
Co-occurrence network analysis of bacteria and fungi in the rhizosphere soil of different halophytes.

The rhizosphere bacterial–fungal co-occurrence network showed that the OTUs in the nodes were mainly composed of bacteria, such as *Acidobacteriota*, *Actinobacteriota*, and *Bacteroidota*, as well as fungi, including *Ascomycota* and *Basidiomycota*. Among them, the *R. soongarica* rhizosphere network had higher connectivity and complexity (nodes = 633, edges = 3300), but the positive/negative correlation of edges was the lowest (21.58%) (Figure 6, Table 2).

### 3.5. The Complexity of Bacterial–Fungal Co-Occurrence Network in the Rhizosphere of Halophytes

**Figure 7:**
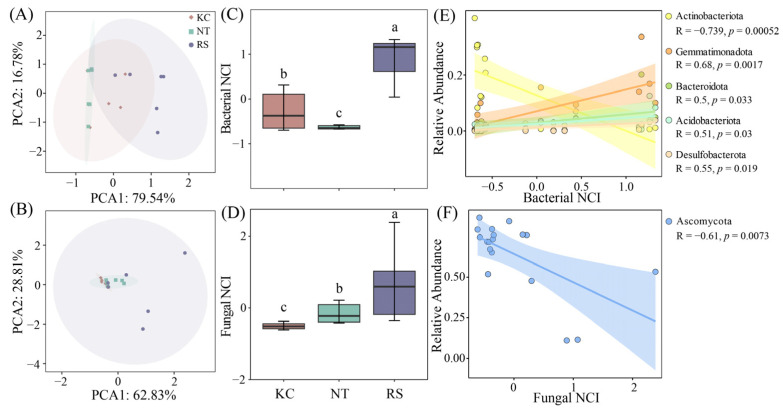
PCA analysis of bacterial and fungal network parameters in the rhizosphere soil of halophytes (**A**,**B**), network complexity indices (**C**,**D**) and linear fitting of dominant phyla (**E**,**F**). Different lowercase letters indicate significant differences.

The complexity of the *R. soongarica* rhizosphere bacterial and fungal networks was higher than that of *K. cuspidatum* and *N. tangutorum* (Figure 7A–D). Actinobacteriota, Gemmatimonadota, Bacteroidota, Acidobacteriota, and Desulfobacterota had a significant linear relationship with the bacterial NCI index, and Actinobacteriota showed a negative correlation (Figure 7E). Among fungi, Ascomycota showed a negative correlation with the fungal NCI index (Figure 7F). In the subnetwork, the network nodes, edges, and average density all showed significant correlations with Na^+^ and TC. The edges, average density, Transitivity, and Average path length also had a relatively obvious correlation with the changes in TP and AN (Figure S3).

**Figure 8:**
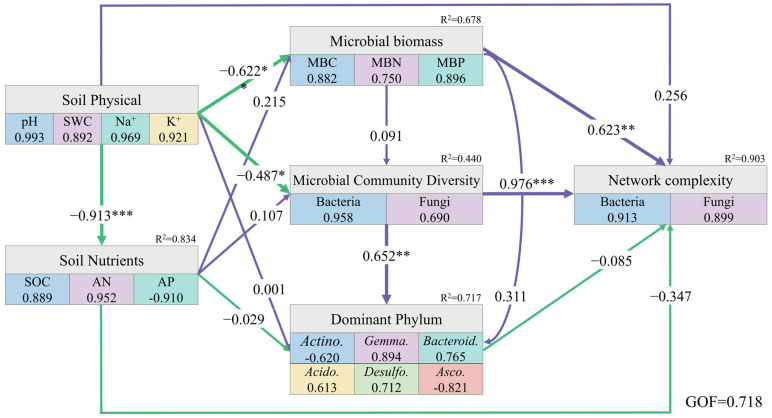
Partial least squares path model of the rhizosphere soil of halophytes. Purple and green arrows indicate positive and negative causality, respectively, the numbers on the arrows indicate normalized path coefficient, R^2^ indicates the variance explained variable explained by the model. (*** *p* < 0.001, ** *p* < 0.01, * *p* < 0.05).

By constructing PLS-PM, the physical properties of the rhizosphere soil in halophytes had a direct negative effect on the chemical properties, while simultaneously negatively regulating microbial biomass and microbial community diversity. The biomass of rhizosphere microorganisms had a significantly positive effect on the network complexity. Moreover, microbial community diversity not only had a direct positive impact on the composition of the dominant phyla but also significantly regulated microbial network complexity (Figure 8).

## 4. Discussion

### 4.1. Differential Rhizosphere Strategies of Halophytes in Desert-Grassland Salt Marsh

The differences in physicochemical properties and microbial biomass among the rhizosphere microenvironments of the three halophytes in the Jiantan wetland reflect their unique ecological adaptation strategies. The rhizosphere of *K. cuspidatum* exhibited the highest pH, SWC, and Na^+^ and K^+^ contents, suggesting that *K. cuspidatum* may have a stronger ion enrichment capacity or selectively retain high concentrations of Na^+^ and K^+^ in the rhizosphere region to maintain osmotic balance for physiological regulation [3]. In contrast, although the Na^+^ and K^+^ content in the rhizosphere of *R. soongarica* is relatively low, it has the highest TC, TOC, and AN content, which provides a more abundant carbon and nitrogen source for its rhizosphere microorganisms [18]. More importantly, the MBC, MBN, and MBP contents in the rhizosphere of *R. soongarica* were significantly higher than those of *K. cuspidatum* and *N. tangutorum*, indicating that its rhizosphere microbial community has a higher biomass and activity. This high microbial biomass may be closely related to the relatively high organic matter content and nutrient availability in the rhizosphere of *R. soongarica*, providing more favorable conditions for the growth and reproduction of microorganisms [44].

### 4.2. Typical Composition of Halophyte Rhizosphere Microbial Communities

In this study, the Shannon and ACE indices of the rhizosphere fungi of *K. cuspidatum* were significantly higher than those of *R. soongarica*, combined with higher salt (Na^+^ and K^+^) and SWC in the rhizosphere. On the one hand, it can be inferred that the rhizosphere fungal community of halophytes is more sensitive to environmental factors [45]; on the other hand, the high-salt environment in the rhizosphere of *K. cuspidatum* may have maintained a relatively high fungal diversity by inhibiting some sensitive fungal groups and screening out rare fungal species with stronger salt tolerance [3,4,5,6,7,8]. In the NMDS analysis, the differentiation of bacterial community structure (NMDS1 axis) was mainly positively driven by salinization factors (pH, Na^+^, K^+^, and TP), whereas organic matter and nitrogen (TC, AN, and MBN) had inhibitory effects. The structure of the fungal community was positively correlated with salt ions (Na^+^ and K^+^) and pH and negatively correlated with carbon sources (TC and TOC). These results suggest that the assembly of bacterial communities may be more directly regulated by salt stress and phosphorus dynamics, whereas fungal communities tend to form differentiated distributions in the trade-off between salt enrichment and the availability of carbon and nitrogen resources, which has been confirmed in previous studies [14,21,46].

The rhizosphere bacterial community of halophytes is mainly composed of *Proteobacteria*, *Firmicutes*, *Actinobacteriota*, and *Gemmatimonadota*. The fungal community was dominated by *Ascomycota*, *Mucoromycota*, and *Basidiomycota*, and this composition pattern is consistent with the characteristics of a typical halophytic environment microbial community [21,47]. More importantly, random forest analysis identified the microbial groups that were most sensitive to changes in the rhizosphere microenvironment of different plants. Their high interpretation rates for changes in physicochemical properties (all exceeding 48%) indicate that these groups are potential key species driving the functional differentiation of rhizosphere microorganisms [48]. *Desulfobacterota* participate in sulfur cycling, and abundance variation may be associated with rhizosphere redox potential and sulfur metabolism [49]. *Mortierellomycota*, an important rhizosphere fungus, its distribution may be strongly influenced by the heterogeneity of rhizosphere carbon and nitrogen resources dominated by plant types [50].

At the level of rhizosphere bacteria in halophytes, the close association between specific groups and environmental factors provides concrete evidence for this rule. For instance, the abundance of genera such as *Bacillus* and *Pseudomonas*, which are common multifunctional bacterial genera in the rhizosphere, is widely related to various environmental factors, demonstrating the flexibility and functional diversity of bacterial communities in response to environmental changes [51,52]. In contrast, fungi such as *Gibberella* and *Alternaria* show specific associations with key salt and nutrient factors such as pH, Na^+^, TC, and AN, which once again confirms the pattern that the fungal community structure is strongly screened by a few key environmental factors [53,54]. Further research indicated that soil pH profoundly influences plant root development and nutrient uptake efficiency by regulating the chemical form and bioavailability of mineral elements, thereby affecting the composition of the rhizosphere microbial community [55]. The TC content in the plant rhizosphere reflected that the allocation of photosynthetic products is the key factor driving spatial heterogeneity in the rhizosphere microbiome [56]. The nitrogen availability can effectively alleviate the N competition in the plant rhizosphere and enhance the decomposition of SOC by the microbial community [57].

### 4.3. Structure, Stability, and Environmental Drivers of the Microbial Interaction Network

This study conducted an in-depth analysis of the rhizosphere bacterial–fungal co-occurrence network of halophytes and found that the rhizosphere of *R. soongarica* has the largest number of nodes and edges and the highest connectivity co-occurrence network, indicating that it supports the most complex and close bacterial–fungal interaction relationship [58]. This high level of network complexity is usually associated with greater functional redundancy and ecosystem stability, which is consistent with the previously observed higher TC, MBC, and MBN in the rhizosphere of *R. soongarica*. Abundant resources provide a basis for the coexistence and interaction of a large number of microbial groups [59]. However, the proportion of negatively correlated connections in the *R. soongarica* network was the lowest, indicating that its complex network structure is mainly driven by a large number of positively correlated interactions (competition or antagonism), forming a more coordinated microbial community [60].

Subnetwork analysis provides clues for understanding the driving factors of this complexity [41]. *Actinobacteriota* and *Ascomycota*, key phyla of bacteria and fungi, respectively, had relative abundances that were negatively correlated with the NCI index. This indicates that they may play the role of “core competitors” or “dominant players” in the community, and the strengthening of their dominant position may simplify the interaction network [17]. In contrast, the positive correlation between groups such as *Gemmatimonadota*, *Bacteroidota*, and NCI suggests that they may be “bridge” groups that enhance network connectivity and complexity [61,62]. In particular, the correlation analysis between network topological attributes and environmental factors clearly outlines the two dominant forces driving the construction of interactive networks, namely, salt stress (Na^+^) and carbon source basis (TC), which jointly affect the scale (nodes, edges) and connection tightness (average density) of the network [46]. The availability of phosphorus (TP) and nitrogen (AN) more directly regulates the advanced organizational characteristics of the network, such as transitivity and average path length, which are closely related to the efficiency and stability of the network [63].

## 5. Conclusions

Overall, the differences in the environment and plant types in the Jiantan wetland jointly shape a unique rhizosphere physicochemical microenvironment, which in turn drives the differentiation of bacterial and fungal communities along different ecological gradients. Although the rhizosphere of halophytes shares a similar microbial phyla background, due to the differences in salt and nutrients, different intensities of ecological screening have been carried out for rhizosphere bacterial and fungal communities. The halophyte *R. soongarica* constructs a cooperative, structurally complex, and functionally highly integrated bacterial–fungal interaction network by creating a relatively low-salt and high-organic rhizosphere environment, which may be an important microbiological mechanism for maintaining the stability of its rhizosphere microecosystem.

## Figures and Tables

**Table 1:** Physicochemical properties of rhizosphere soil of halophytes.

	KC	NT	RS
pH	8.81 ± 0.04 **a**	8.47 ± 0.05 **b**	8.29 ± 0.06 **c**
SWC	27.76 ± 1.08 **a**	21.92 ± 0.86 **b**	19.76 ± 0.2 **b**
Na^+^ (mg/kg)	5728.9 ± 451.97 **a**	1384.43 ± 122.46 **b**	580.15 ± 35.26 **b**
K^+^ (mg/kg)	58.16 ± 4.47 **a**	34.49 ± 1.85 **b**	28.28 ± 1.9 **b**
TC (g/kg)	6.91 ± 0.6 **c**	10.77 ± 0.77 **b**	13.34 ± 0.27 **a**
TOC (g/kg)	2.93 ± 0.21 **b**	5.49 ± 0.44 **a**	6.37 ± 0.62 **a**
TN (g/kg)	0.25 ± 0.01 **b**	0.49 ± 0.1 **a**	0.35 ± 0.03 **ab**
TP (g/kg)	0.34 ± 0.02 a**b**	0.37 ± 0.03 **a**	0.29 ± 0.02 **b**
AN (mg/kg)	6.1 ± 0.3 **c**	9.26 ± 0.42 **b**	11.52 ± 0.58 **a**
OP (mg/kg)	0.91 ± 0.03 **a**	0.6 ± 0.05 **b**	0.54 ± 0.03 **b**
MBC (mg/kg)	38.92 ± 1.69 **b**	45.72 ± 0.76 **a**	51.16 ± 2.23 **a**
MBN (mg/kg)	2.56 ± 0.08 **b**	2.74 ± 0.06 **b**	3.22 ± 0.24 **a**
MBP (mg/kg)	0.72 ± 0.02 **b**	0.87 ± 0.02 **a**	0.91 ± 0.03 **a**

Note: Different lowercase letters indicate significant differences.

**Table 2:** Network parameters in the rhizosphere soil of halophytes.

	KC	NT	RS
Bacterial nodes	424	273	574
Fungal nodes	6	95	59
Edges	2392	2996	3300
Positive edges	2330	2934	3154
Negative edges	62	62	146
Modularity	0.69	0.40	0.64
Number of modules	21	28	18
Average path length	6.57	4.72	5.84
Graph diameter	16.82	16.01	15.93
Graph density	0.02	0.04	0.02
Clustering coefficient	0.56	0.59	0.51
Betweenness centralization	0.15	0.05	0.04

## Data Availability

No new data were created or analyzed in this study.

## Supplementary Materials

The following supporting information can be downloaded at: https://www.mdpi.com/article/10.3390/microorganisms14030635/s1, Figure S1. The top 10 bacterial genera in the rhizosphere soil of halophytes. Different lowercase letters indicate significant differences. Figure S2. The top 10 fungal genera in the rhizosphere soil of halophytes. Different lowercase letters indicate significant differences. Figure S3. The correlation between the network parameters of bacteria and fungi in the rhizosphere soil of halophytes and their physicochemical properties. (*** *p* < 0.001, ** *p* < 0.01, * *p* < 0.05).
