# Supplementary material for: Halophyte-Specific Rhizosphere Effects Drive the Differentiation of Microbial Community Assembly in a Desert-Grassland Salt Marsh
Source: Microorganisms. 2026 Mar 11;14(3):635. doi: 10.3390/microorganisms14030635 (PMC13029671; doi:10.3390/microorganisms14030635)
Supplement: Supplementary file 1 [file microorganisms-14-00635-s001.zip › microorganisms-4140703-supplementary.pdf]

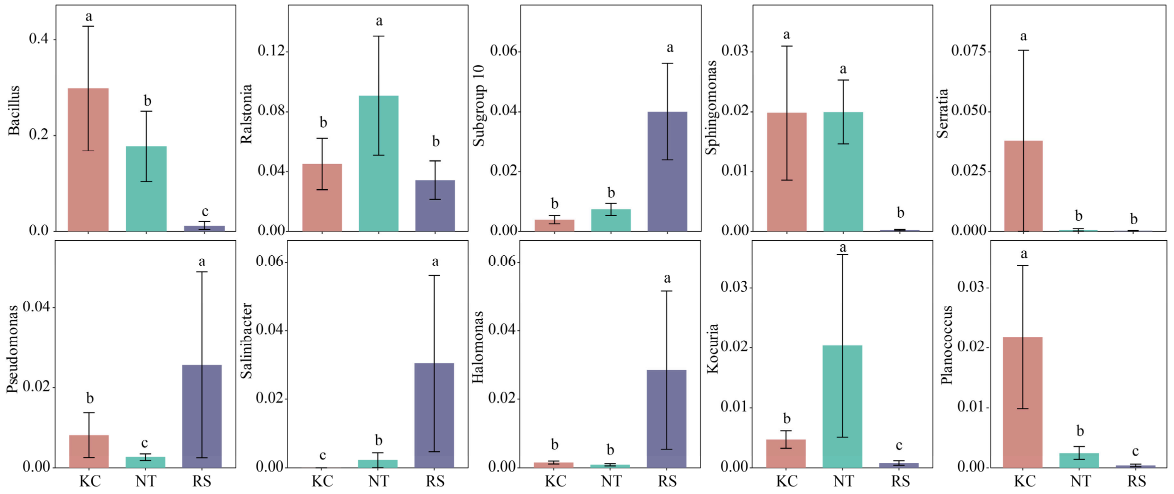

Figure S1. The top 10 bacterial genera in the rhizosphere soil of halophytes. Different lowercase letters indicate significant differences.

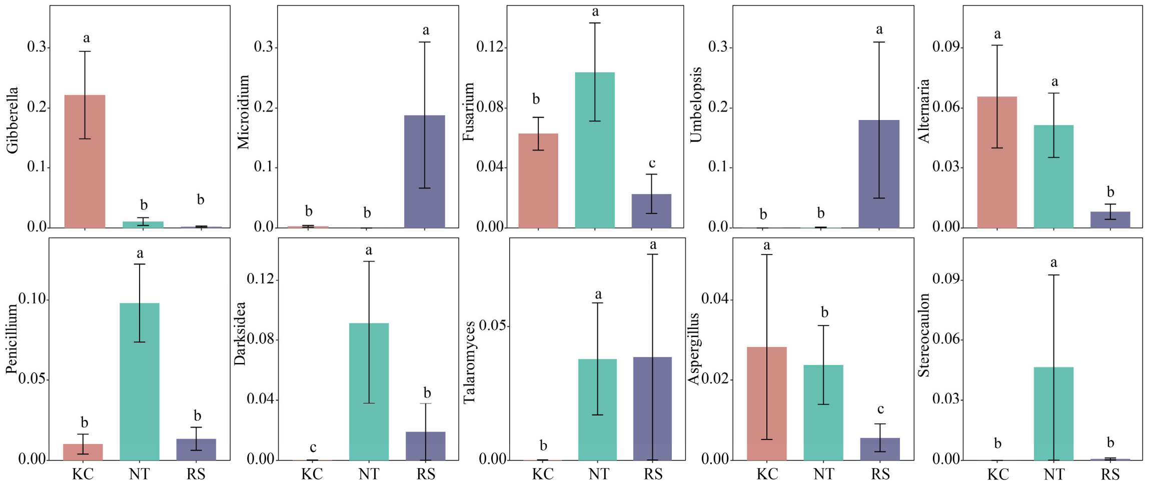

Figure S2. The top 10 fungal genera in the rhizosphere soil of halophytes. Different lowercase letters indicate significant differences.

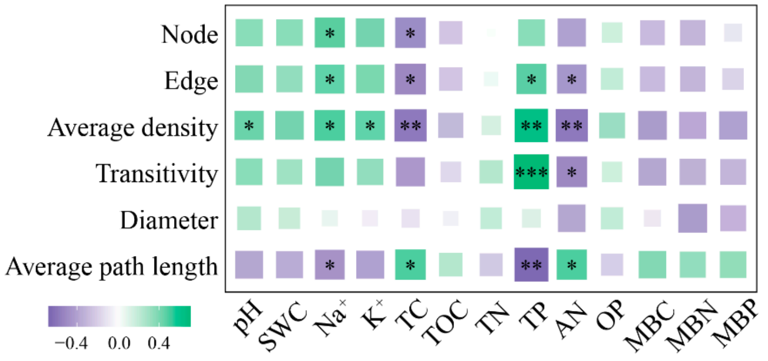

Figure S3. The correlation between the network parameters of bacteria and fungi in the rhizosphere soil of halophytes and their physicochemical properties. (*** $p < 0.001$, ** $p < 0.01$, * $p < 0.05$).
